# A free vascularized tibia-fibular composite graft for the traumatic femoral bony defect of a 6-year-old boy with 10-year follow up: a case report

**DOI:** 10.1186/1752-1947-7-136

**Published:** 2013-05-28

**Authors:** Shih-Hao Chen, To Wong, Ming-Chung Yeh, Chun-Huang Pai, Jih-Yang Ko

**Affiliations:** 1 Department of Orthopedics, Tzu Chi General Hospital at Taichung and Tzu Chi University, Taichung, Taiwan; 2 Department of Orthopedics, Kaohsiung Municipal Hospital, No.976, Jhonghua 1st Rd., Gushan Dist., Kaohsiung City 804, Taiwan; 3 Department of Plastic Surgery, Kaohisung Chang Gung Memorial Hospital, Kaohsiung, Taiwan; 4 Department of Orthopedics, Cishan Hospital, Department of Health, Cishan, Taiwan; 5 Department of Orthopedics, Kaohisung Chang Gung Memorial Hospital, Kaohsiung, Taiwan

**Keywords:** Pediatrics, Traumatic bony defect, Vascularized tibia-fibular graft

## Abstract

**Introduction:**

Free vascularized fibular grafts have been widely used for the reconstruction of long bone defects. However, the use of a vascularized tibial graft is precluded by its weight-bearing function and unacceptable donor site morbidity.

**Case presentation:**

We present a rare case of using a vascularized tibia-fibular composite graft taken from a 6-year-old Chinese boy’s ipsilateral lower leg to reconstruct a large bony defect of his traumatic femur. Hypertrophy of the tibial graft, good remodeling of the femoral shaft, and atrophy of the unloaded fibular graft were noted at the 10-year follow up. He was able to participate in outdoor activities such as basketball while wearing his prosthesis.

**Conclusions:**

The 10-year follow up demonstrates the feasibility of this salvage procedure for a floating knee injury with neurovascular compromise.

## Introduction

Since Taylor [1] reported the first case of vascularized bone graft to reconstruct a large defect in a tibia in 1977, free vascularized fibular grafts have been widely accepted for the reconstruction of long bone defects, especially when the defect is greater than 6cm [1-10]. However, the management of large long-bone defects with autogenous vascularized tibial bone is precluded by its weight-bearing function and unacceptable donor site morbidity. In selected cases, for example when a chronic femoral non-union persists after other reconstructive procedures, the tibia might provide an alternative as long-bone reconstruction donor tissue [2,11,12]. We present the case of a 6-year-old child in whom a free vascularized tibia-fibular composite graft taken from the amputated ipsilateral leg was used to reconstruct an acute 9cm bony defect of the femur. To the best of our knowledge, this is the first report of a transfer of a free vascularized tibia-fibular composite graft to reconstruct an acute traumatic defect of the femur with neurovascular compromise in a long-term follow up.

## Case presentation

**Figure 1:**
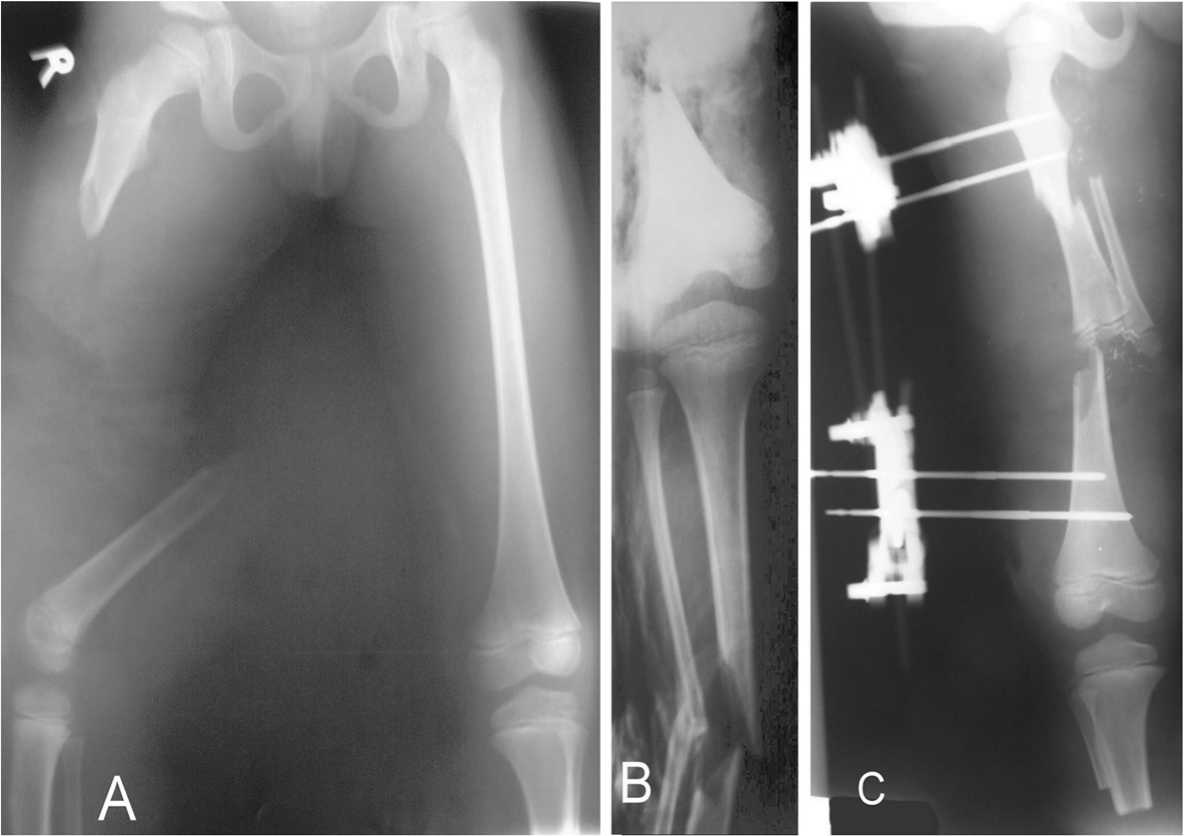
**Radiograph of initial managment. A**: Initial film post-injury demonstrates the right femoral shaft fracture with a 9cm bony defect. **B**: The right tibia and fibula also had comminuted, Gustilo type IIIC fractures that necessitated a below-knee amputation. **C**: A free vascularized tibia-fibular composite graft was used to reconstruct the femoral defect.

A 6-year-old Chinese boy sustained a crush injury in a motor vehicle accident resulting in extensive damage to his right lower limb with comminuted fractures of his right femur and his right tibia-fibula. His right femur was a Gustilo type IIIC fracture with sciatic nerve and femoral artery tears associated with a 9cm bony fragment lost during the trauma (Figure 1A). His right tibia-fibula also had severely comminuted, Gustilo type IIIC fractures (Figure 1B). He was taken to the operation room 5 hours after injury. A right below-knee amputation was performed on the basis of the major neurovascular damage to the femoral artery, vein and sciatic nerve in his thigh along with the severe soft tissue injury and comminution of his distal tibia-fibula. The amputated specimen was prepared with normal saline irrigation. The distal tibia-fibular osteomuscular flap was then harvested to microsurgically reconstruct the femoral bony defect. Informed consent to perform this procedure and report the case was obtained from the patient and his parents. Due to segmental loss of the femoral artery and vein, the tibia-fibular osteocutaneous flap pedicle was anastomosed with a flow-through artery and vein graft. The posterior tibial artery was anastomosed to the torn femoral artery, and the posterior tibial vein was anastomosed to a superficial vein in the groin area. The femoral vein was grafted using the greater saphenous vein from the amputated specimen. The sciatic nerve was repaired directly using 9–0 nylon end-to-end perineurium repairs. Ankle joint cartilage was removed to increase the contact area for the incorporation of the distal femur-graft. A Hoffmann’s external fixation was applied to keep the same length as the opposite leg and immobilize the fracture (Figure 1C). After meticulous debridement, the wound was sutured directly without any skin defect, because adequate skin and soft tissue were provided by the tibia-fibular composite graft. The operation took 6 hours and 40 minutes.

Postoperatively, the patient was treated with intravenous antibiotics using cefazolin 500mg every 8 hours and gentamicin sulfate 20mg every 8 hours. No anticoagulants were used postoperatively in this patient. Subsequent wound cultures grew *Escherichia coli* and *Streptococcus bovis*. On the 7th postoperative day, bone scanning using technetium-labeled methylene diphosphonate was performed which demonstrated positive uptake on both ends of the donor tibia-fibular graft. Because the right leg was not stable enough, no specific physical therapy or temporary prosthesis was used. The wound at the medial and posterior aspect of the right thigh became erythematous and purulent discharge was noted 11 days after the initial operation. Debridement of the necrotic muscle and tissue was performed and the wound was then closed loosely. The antibiotics were changed to cefamandole 250mg every 8 hours and ampicillin 500mg every 6 hours, based on the culture data. The stump of the below-knee amputation also became necrotic and was debrided 26 days after the initial surgery. The skin and wound condition were stabilized thereafter.

**Figure 2:**
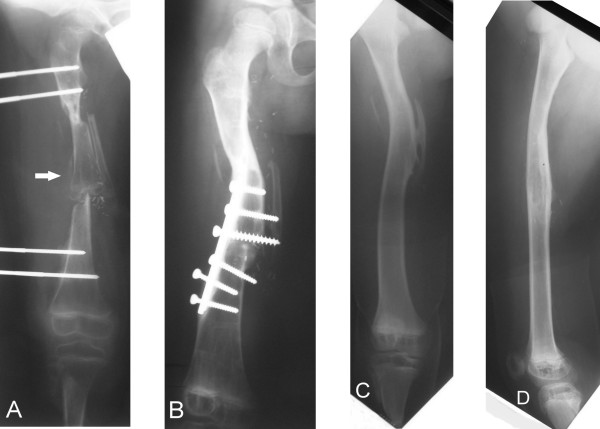
**Radiograph of treatment progression. A**: Radiograph at 4 months after the initial operation showed delayed callus formation (arrow) and pin tract infection at the distal femur. There appears to be bony union proximally between the graft and the proximal femur. Some longitudinal growth of the transferred tibia was also noted. At that time, a change was made from Hoffmann’s external skeletal fixation to an internal compression plate supplemented by an autogenous iliac bone graft. **B**: Radiograph at 12 months after the initial operation revealed complete incorporation of the vascularized tibia-fibular bone grafts and 25 degrees varus angulations in the femur. The deformity was noted 10 months after the initial operation, probably due to screw loosening. **C** and **D**: Plain radiographs obtained 10 years after surgery. Note atrophy of the unloaded fibular graft and remodeling of the tibial graft.

**Figure 3:**
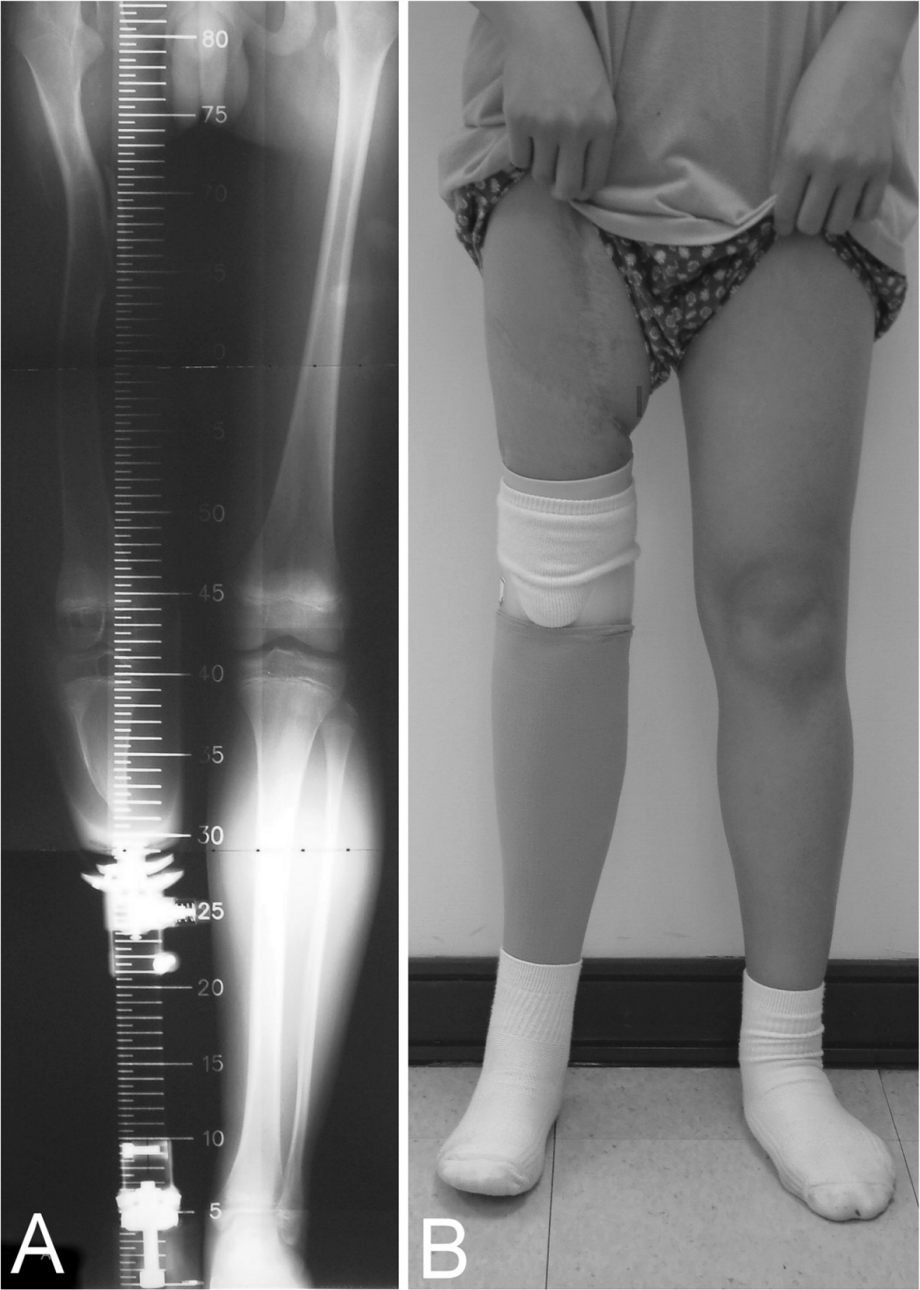
**10 years follow-up presentation. A**: Standing scanogram at 10 years after surgery. **B**: Clinical photo of the patient with prosthesis.

The patient was discharged 6 weeks after the operation, with the wound clear and the infection under control. Non-weight-bearing crutch walking was started 5 weeks after the surgery and partial-weight-bearing was allowed 2 months later when the patient used 50% weight-bearing with the aid of a below-knee prosthesis. Unfortunately, delayed callous formation at the distal femoral-graft junction and pin tract infection in the distal femur were noted at 4 months after surgery (Figure 2A). At that time, a change was made from the Hoffmann’s external skeletal fixation to an internal compression plate supplemented by an autogenous iliac crest bone graft. During the operation, the pin holes of the external fixation device were curetted and the iliac bone graft was put on the distal end of the host-donor bones. The transferred epiphyseal plate of the distal tibia and fibula were removed because there was no length discrepancy of the injured femur at 4 months. The pin tract infection healed 2 weeks after the removal of the Hoffmann’s external skeletal fixation. Over the subsequent 6 months, serial radiographs revealed complete incorporation and gradual remodeling of the vascularized tibia-fibular composite graft in the femur (Figure 2B). Walking with full weight-bearing was allowed at 8 months after the surgery. Plate removal was performed 1.5 years after the operation due to chronic irritation from the loosened plate and screws, and for fear that the plate might hinder further remodeling and hypertrophy of the femur. Hypertrophy of the tibial graft, good remodeling of the femoral shaft, and atrophy of the unloaded fibular graft were noted at the 10-year follow up (Figures 2C and D). On the standing scanogram, both the hip and the knee joints were at the same level, and the femurs were of equal length (Figure 3A). Clinically, there were symmetrical alignments of both legs. The boy did well with his prosthesis, including all his daily activities. He was able to participate in outdoor activities such as basketball while wearing his prosthesis (Figure 3B). The range of motion of the right knee was between 5 and 95 degrees in extension–flexion and that of the right hip was between 0 to 90 degrees in extension–flexion, 40 degrees in abduction, 15 degrees in adduction, 15 degrees in external rotation, and 10 degrees in internal rotation. The repaired sciatic nerve was functioning well, in that there was full strength of hip extension and knee flexion indicating good function of the hamstring muscles and normal sensation at the stump.

## Discussion

Severe open fractures are surgical emergencies that should be thought of as incomplete amputations [13]. The only absolute indication for amputation in trauma is an ischemic limb with a non-reconstructible neurovascular injury. In the lower extremity, a severe open fracture (Gustilo–Anderson grade IIIC) combined with an insensate plantar foot surface is generally treated with an immediate amputation. It was for these reasons we initially performed an immediate below-knee amputation for our patient. Instead of performing an above knee amputation, we tried to preserve the knee joint so that this young patient would have better function and activity of his right lower extremity later in life. Our concerns at the time of operation were whether the transferred epiphyseal plate of the distal tibia would affect ultimate femoral length, the fate of the transferred unloaded fibula, the neurovascular condition of the salvaged limb, and knee function. The challenges during surgery and the perioperative period included the reconstruction of limb length, alignment, host-donor junctional union, neurovascular function, and weight-bearing capacity to avoid stress fracture and subsequent infection. Through successful microsurgery, together with bony transfer, neurovascular anastomosis, soft tissue coverage, sequential debridement and effective parenteral antibiotics usage, we were able to salvage the lower extremity and eradicate the skeletal infection with favorable results. However, due to a lack of rigid fixation, delayed callus formation and pins loosening, we changed the Hoffmann external fixation to plate fixation with an autogenous local bone graft at 4 months after surgery. The rigid internal fixation improved the bony incorporation between the donor and host bone junction, maintained correct alignment of the limb, and promoted the union rate at 8 months after the initial injury. The improved blood supply after the vascularized flap also helped overcome the skeletal infection.

Free vascularized fibular grafting has been reported many times in the literature for use in the salvage of massive bony defects, and that hypertrophy of the free vascularized fibular graft can also increase the limb’s weight-bearing capacity [3,4,6-10,14]. However, there have been no published reports regarding the free vascularized distal tibia-fibular graft. The considerations in our decision to use the amputated specimen as the osteomuscular graft include the following: (1) a 9cm femoral bony defect was difficult to reconstruct using other methods; (2) sciatic nerve regeneration might not reach the ankle even after successful repair; and (3) unpredictable limb length discrepancy and soft tissue scarring will influence knee function in the future. Several options for reconstructing a massive bony defect in a floating knee injury with neurovascular compromise might include: (1) vascularized allograft [6]; (2) vascularized fibula from the amputated specimen or from the contralateral leg [3-5,7-10]; (3) acute shortening; and (4) Ilizarov’s bone-transfer and lengthening [14-19]. In this specific situation, however, a free vascularized tibia-fibula autogenous bone graft was thought to be the best method to decrease the morbidity, achieve sufficient weight-bearing support, prevent the possibility of rejection or transmissible diseases from an allograft, and preserve the knee function. Because the length of the distal tibia-fibular amputee was relatively shorter than the length of the femoral bony defect, we preserved the distal tibial epiphyseal plate to prevent the graft from becoming too short in the future. A similar procedure, in which a vascularized transplant of the proximal fibula was performed with an open physis to allow the growth potential of the segment, was reported by Ceruso *et al*. [11]. The transferred epiphyseal plate in our patient was removed at 4 months (during a plate exchange with the original external fixator), because there was no length discrepancy between the bilateral femurs at that time. Some alternative forms of fixation between the femur and the tibial graft, such as transfixing pins into the host-donor bones, might help stabilize the fractured site, keep the alignment, and allow application of the external skeletal fixation until bony union. However, relapsed skeletal infection and an unequal limb length must be avoided.

For a segmental bone gap of 6cm or less, traditional bone grafting techniques may suffice. For a defect greater than 6cm, a fibula is preferable as a free vascularized bone graft [3-5,8-10] because of its length, longitudinal configuration, limited donor site morbidity, and predictable vascular pedicle for limb reconstruction, although Ilizarov’s lengthening is another option [14-19]. Free vascularized fibular grafting has the following advantages: (1) It accomplishes neurovascular anastomosis and limb length in a one-stage procedure. (2) The graft may be doweled into the position proximally and distally for femoral stability during reconstruction. (3) Bleeding bone with endosteal and periosteal circulation is transferred to the recipient site. (4) Vascularity of the periosteum influences the degree of hypertrophy more in the medullary cavity of the femur than in the onlay graft. (5) If the anastomosis fails, the vascularized bone may function as a traditional cortical bone graft [3-5,9,10,20]. Despite the distinct advantages of a free vascularized fibular graft, this procedure cannot correct limb length discrepancy, which is the main advantage of the Ilizarov’s bone-transfer method [14-18]. Angular deformity, particularly observed in the fracture of the upper or lower third of the femur because of the angular muscle forces, needs preoperative skeletal traction, intraoperative adjustment and rigid fixation of the leg length and alignment. We found similar advantages in our vascularized tibia-fibular composite graft and larger cross-sectional area for mechanical support. Besides, in the situation of a floating knee injury with severe neurovascular compromise, the use of vascularized tibia-fibular osteomuscular grafting is an optimal choice for reconstructing the injured lower limb and improving functional outcomes.

Delayed callus formation was found in our patient, because we initially used Hoffmann’s external fixation for a leg that sustained a high-energy trauma with potential skeletal infection. We were concerned whether the transferred distal tibial epiphyseal plate would influence the ultimate length of the femur, alignment, and host-donor bony union. The second-staged operation using dynamic compression plating supplemented by an iliac bone graft rigidly aligned the host-donor bones and achieved early union. The transferred epiphyseal plate of the distal tibia and fibula were removed at that time because there was no length discrepancy of the injured femur. On the standing scanogram at the 10-year follow up, the limb proved to be on the same axis as the normal leg, and the knee and hip joints were both at the same level (Figures 3A and 3B). Although the patient was only 16 years old, we thought there would be no femoral length discrepancy in the future because both femoral epiphyseal plates were intact and equal in closure to the contralateral normal lower extremity. The patient is satisfied with the result and has an acceptable walking ability.

## Conclusions

To the best of our knowledge, the transfer of a tibia-fibular graft containing the zone of injury from high-energy trauma to reconstruct a defect in the ipsilateral femur has not been reported. In our case, the alignment and length of the remodeled femur were maintained due to the successful microsurgery and secondary rigid fixation. The long-term result demonstrates the feasibility of using a microvascular transfer of a vascularized tibia-fibular graft to reconstruct a massive femoral bony defect in a severe floating knee injury with neurovascular compromise.

## Consent

Written informed consent was obtained from the patient’s legal guardians for publication of this case report and accompanying images. A copy of the written consent is available for review by the Editor-in-Chief of this journal.

## Competing interests

The authors declare that they have no competing interests.

## Authors’ contributions

S-HC participated in the case management and was a major contributor in writing the manuscript. TW participated in data collection, literature review and draft writing. M-CY participated in the surgical management of vascularized flap reconstruction. C-HP and J-YK participated in the trauma management of fracture and surgical consultation. All authors read and approved the final manuscript.
